# Transvenous Lead Extraction Using a Stepwise Bailout Strategy: A Single-Center Experience with Wrap-Around Rescue in Selected Bailout Cases

**DOI:** 10.3390/jcm15176832

**Published:** 2026-09-03

**Authors:** Ebru Şahin, Hakan Aksoy, Cansu Selcan Akdeniz, Begüm Yetiş Sayın, Sercan Okutucu, Ali Oto

**Affiliations:** 1 Department of Cardiology, Memorial Ankara Hospital, Çankaya, 06520 Ankara, Türkiye; haksoy63@gmail.com (H.A.); scansuakdeniz@gmail.com (C.S.A.); begumyts@yahoo.com (B.Y.S.); alioto@tksv.org (A.O.); 2 Department of Cardiology, Lokman Hekim University Ankara Hospital, Sincan, 06936 Ankara, Türkiye; sercanokutucu@gmail.com

**Keywords:** transvenous lead extraction, cardiac implantable electronic device, lead dwell time, femoral bailout, snare, wrap-around technique

## Abstract

**Background/Objectives:** Femoral techniques can rescue transvenous lead extraction (TLE) when the superior approach is insufficient, but wrap-around rescue within a stepwise pathway remains incompletely characterized. We described a single-center cohort and examined the association between target-lead dwell time and bailout use. **Methods:** Among 82 retrievable TLE records from 2014 to 2025, four image-only records lacked the clinical, procedural, and follow-up information required for analysis; 78 patients were included. Bailout comprised femoral gooseneck snaring and/or RF Marinr catheter wrap-around. **Results:** Complete procedural success was 77/78 (98.7%), lead-level complete removal 155/156 (99.4%), clinical procedural success 78/78 (100.0%), bailout complete removal 18/19 (94.7%), and wrap-around complete removal 16/17 (94.1%). Median extraction-phase and total skin-to-skin durations were 79.7 and 110.1 min, respectively; median extraction fluoroscopy time was 21.8 min and system-reported air kerma 324.5 mGy. Bailout procedures had longer fluoroscopy than primary-only procedures (31.4 vs. 16.6 min; exploratory *p* = 0.011), whereas extraction-phase duration and air kerma did not differ significantly. Formal major and minor complications were 0/78 through day 30; four minimal subcutaneous hematomas required observation only. In an exploratory Firth model, dwell time remained associated with bailout (adjusted OR/year, 1.14; 95% CI, 1.05–1.25; *p* < 0.001). **Conclusions:** Wrap-around was used within a successfully selected bailout pathway, but its independent effect cannot be isolated. Resource and association analyses are exploratory and noncausal.

## 1. Introduction

The marked increase in the use of cardiac implantable electronic devices (CIEDs) in recent years has been accompanied by a parallel rise in CIED-related complications, including infection, lead dysfunction, lead-related tricuspid valve dysfunction, venous obstruction, and the need for system upgrades. Accordingly, TLE is now an established treatment strategy in contemporary electrophysiology practice [1,2,3].

Contemporary TLE is associated with high clinical success rates; however, selected procedures remain technically demanding and may carry a greater risk of procedure-related complications. Longer lead dwell time, a greater number of targeted or abandoned leads, passive-fixation mechanisms, and certain ICD lead characteristics have been associated with procedural complexity. Among these factors, lead dwell time is one of the most consistent correlates of escalation beyond simple traction, while recent data suggest that lead type also influences the number and type of extraction tools required as lead age increases. Preprocedural planning is therefore particularly important in patients with fractured leads, inaccessible lead ends, or venous occlusion [2,4,5,6,7].

Femoral tools have long been used when extraction through the implant vein is unsuccessful, and early experience emphasized access to both superior and femoral techniques [8,9]. Subsequent reports have described catheter-snare loops, tandem control, countertraction, and combined superior-femoral approaches [1,7,10,11,12]. These mechanical principles are established. The incremental contribution of the present study is a record-availability-based, single-operator cohort in which wrap-around was used within a stepwise bailout pathway, not the invention of a new catheter-snare principle.

We aimed to describe a single-center, single-operator analytic cohort, focusing on procedures in which wrap-around was selected within a stepwise bailout pathway. We report pathway-level complete removal, lead-level outcomes, consensus-aligned success classifications, prespecified safety windows, and an exploratory penalized model of bailout use. The study was not designed to establish superiority or the independent effect of any component technique.

## 2. Materials and Methods

### 2.1. Study Design and Patient Population

We conducted a retrospective, single-center observational analysis in a small, single-operator TLE program at a private tertiary hospital with on-site cardiac surgical backup. Following a change in the institutional archiving system, complete retrievable records were available for 82 adults who underwent TLE between January 2014 and December 2025. Four records contained procedural images but lacked the clinical history, detailed procedural and material documentation, and follow-up required for analysis; these patients were excluded, leaving 78 in the analytic cohort. The cohort is therefore record-availability based and is not described as consecutive. The center performs approximately 10 TLE procedures per year; complete center-wide annual counts for the entire study period were unavailable and were not used as an analytic denominator.

**Figure 1:**
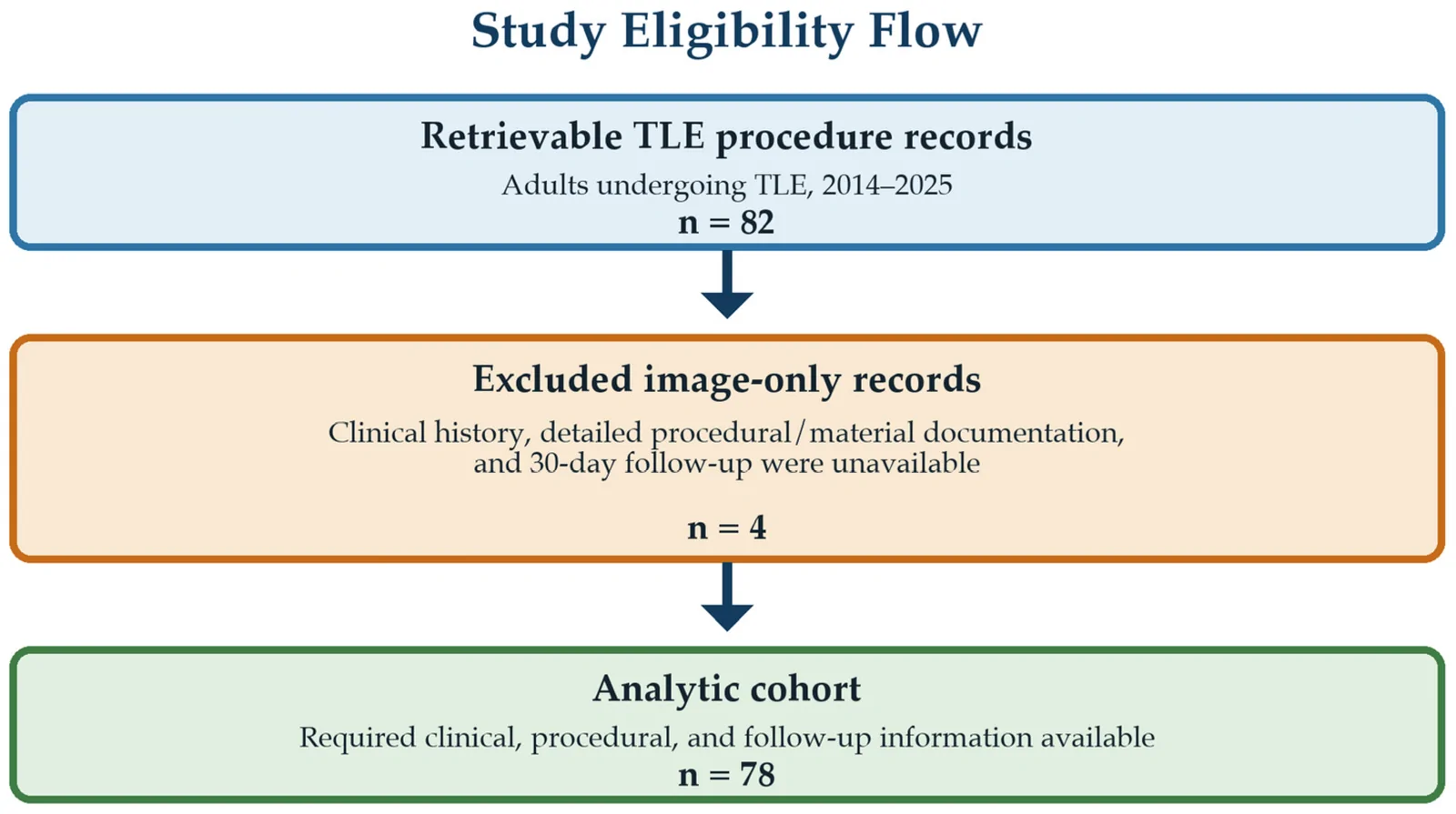
Study eligibility flow diagram. Records were retrievable for 82 adults undergoing TLE during 2014–2025. Four image-only records lacked the clinical history, detailed procedural/material documentation, and follow-up required for analysis; 78 patients were included.

Of 82 patients with retrievable procedure records, four image-only records were excluded because clinical, procedural/material, and 30-day follow-up information was unavailable; 78 patients were analyzed (Figure 1).

Target-lead dwell time was calculated in integer years as extraction year minus the recorded implantation year of the procedural target lead. In bailout procedures, the target was the lead that prompted femoral escalation; in primary-only procedures, it was the lead or leads responsible for the extraction indication. One patient-level dwell value was retained per procedure. When two target leads were present, both had been implanted in the same calendar year and had identical dwell time, so the single value represented both leads. A value of 0 years indicated implantation and extraction within the same calendar year and did not imply zero elapsed days. Target-lead dwell was available for all 78 patients.

The retrospective review and analysis of de-identified clinical data were approved by the Ethics Committee of Ankara Memorial Hospital (decision no. 202607-02-01; 22 July 2026). The study was conducted in accordance with the Declaration of Helsinki and its later amendments. The requirement for individual informed consent was waived owing to the retrospective study design and the analysis of de-identified data. No personally identifiable patient information is reported.

### 2.2. Indications for Lead Extraction

The decision to perform transvenous lead extraction was made by the treating electrophysiology team according to the clinical circumstances at the time of each procedure. For the present analysis, extraction indications were retrospectively reviewed and classified in accordance with the 2017 HRS and 2018 EHRA expert consensus documents [13,14]. Indications included cardiac implantable electronic device infection, lead dysfunction or fracture, venous stenosis or occlusion, and lead-related tricuspid valve dysfunction. Device infection included local pocket infection or erosion and systemic infection, including lead-related infective endocarditis. Lead dysfunction was defined as a clinically significant abnormality in sensing, pacing, impedance, or lead integrity that required lead revision or replacement.

In patients with device-related infection, the generator and all transvenous leads were removed. In noninfectious cases, extraction was initially directed at the lead or leads responsible for the clinical indication. Functioning leads were preserved and reused whenever clinically appropriate. If an additional lead was damaged during the extraction procedure and could no longer be safely retained, it was also removed.

### 2.3. Lead Extraction Procedure and Stepwise Bailout Strategy

All procedures were performed by the same operator under fluoroscopic guidance. Initial anesthesia was local anesthesia with procedural sedation in all 78 cases; no conversion to general anesthesia was recorded. Surgical rescue capability, blood products, and pericardiocentesis and chest-drainage equipment were available; transesophageal/intracardiac echocardiography, invasive arterial monitoring, and femoral arterial access were used or considered according to perceived procedural risk. Femoral venous access was established at the outset when a combined or bailout strategy was anticipated. Preprocedural planning considered lead dwell time, ICD or SVC coils, the number and condition of leads, abandoned leads, venous obstruction, prior unsuccessful extraction, and patient habitus. All index device implantations had been performed at outside institutions; therefore, historical lead model, active-versus-passive fixation mechanism, and ICD coil-configuration data could not be recovered consistently and were not inferred.

After device interrogation and sterile preparation, the generator pocket was opened, the leads were disconnected, and each fixation sleeve was fully released. An active-fixation helix was retracted when feasible. A locking stylet (Cook Medical, Bloomington, IN, USA) or Lead Locking Device (LLD; Spectranetics/Philips, Colorado Springs, CO, USA) was advanced as distally as possible, and the lead body was reinforced with a locking suture or compression coil before controlled traction. A Bulldog Lead Extender (Cook Medical, Bloomington, IN, USA) was used when a cut or fractured lead could not be controlled through its lumen. If controlled traction remained insufficient, a non-powered sheath or a Cook mechanical dilator sheath was advanced while maintaining sheath–lead coaxiality and countertraction. The Evolution Mechanical Dilator Sheath Set (Cook Medical, Bloomington, IN, USA) was used in 2014; from 2015 onward, the Evolution RL Controlled-Rotation Dilator Sheath Set (Cook Medical, Bloomington, IN, USA) was used. Laser sheaths were not used.

The procedural reports, material records, and fluoroscopic images were reviewed for all 19 bailout procedures. The source review was not independently blinded; apparent discrepancies were checked against the contemporaneous records, and unresolved items were retained as unavailable rather than inferred. The institutional sequence began with superior lead preparation, controlled traction, and advancement of a mechanical dissection sheath. Superior extraction continued while a stable coaxial rail and controlled sheath progression could be maintained. The review identified five qualitative reasons for femoral escalation: sheath non-progression; loss of a stable coaxial superior rail; lead deformation, elongation, or impending fracture; lead fracture with loss of superior control of the distal segment; or a judgment that further superior traction would be unsafe. More than one reason could coexist. No fixed time, force, or attempt threshold was used. Fragments that separated during the superior phase but remained retrievable with superior traction were removed before the residual target segment was addressed through the femoral route.

All 19 bailout procedures used the right femoral vein and a 10 Fr venous introducer. Mechanical dissection-sheath diameter was selected according to target-lead type: 9 Fr for atrial and coronary-sinus (left-ventricular) leads and 11 Fr for right-ventricular pacing or shock-coil ICD leads; procedures targeting both categories used both sizes as required. When a capturable free lead end or fragment was present, direct capture was attempted with a 15 mm Amplatz Goose Neck snare (GN1500; Medtronic, Minneapolis, MN, USA). When no free end was available, or direct capture failed or did not provide stable inferior control, a 7 Fr, 110 cm RF Marinr steerable multi-curve ablation catheter (REF 075302; Medtronic, Minneapolis, MN, USA) was advanced from the right femoral vein for wrap-around. A Needle’s Eye Snare was not used.

In all 17 wrap-around procedures, the RF Marinr catheter was passed around the target-lead body in the free right atrium above the tricuspid valve. In each of the 16 combined procedures, the Goose Neck snare captured the distal tip of the Marinr catheter to close the loop; inferior control was then used to mobilize and release the lead, after which the Goose Neck recaptured the freed lead segment for final femoral retrieval. In the single wrap-around-only procedure, stable Marinr control permitted the lead to be drawn directly into the 10 Fr femoral introducer without snare use. Orthogonal fluoroscopic views were used to confirm the relationship of the loop to the target lead and to avoid the tricuspid apparatus. The maneuver was not used for uncontrolled rotational stripping of adhesions.

**Figure 2:**
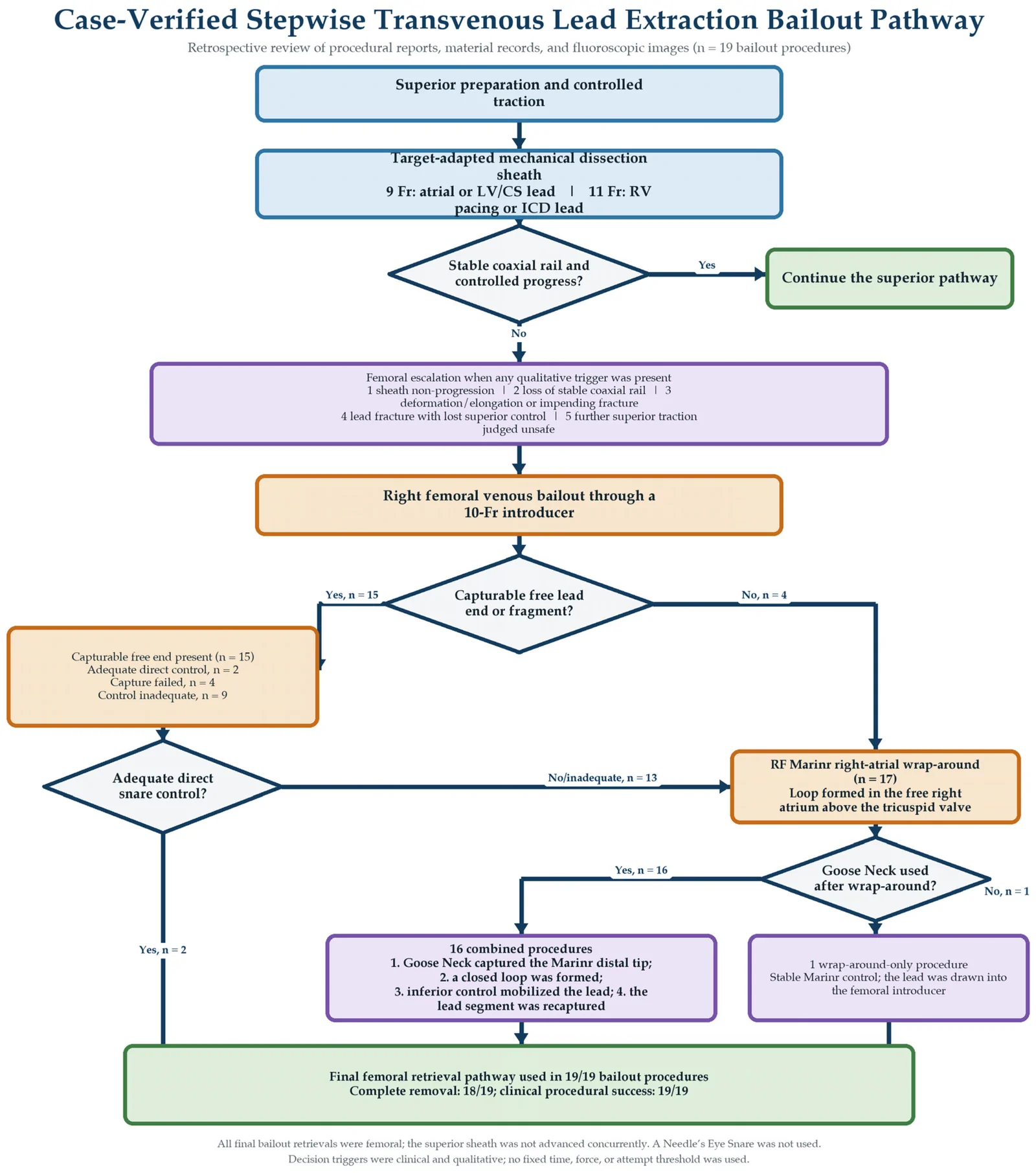
Case-verified stepwise transvenous lead extraction bailout pathway. The flowchart integrates retrospective review of procedural reports, material records, and fluoroscopic images from all 19 bailout procedures. Superior extraction continued while a stable coaxial rail and controlled progress were maintained. Any of five qualitative findings supported right-femoral escalation through a 10 Fr introducer. Direct 15 mm Amplatz Goose Neck capture was attempted when a free end was present; absent, failed, or mechanically inadequate capture led to RF Marinr wrap-around in the free right atrium above the tricuspid valve. In combined procedures, the Goose Neck captured the Marinr distal tip to close the loop, inferior control mobilized the lead, and the freed lead segment was recaptured for femoral retrieval. One wrap-around-only procedure was completed by drawing the lead into the femoral introducer under stable Marinr control. The final femoral retrieval pathway was used in 19/19 bailout procedures; complete removal was achieved in 18/19 and clinical procedural success in 19/19. A Needle’s Eye Snare was not used.

**Figure 3:**
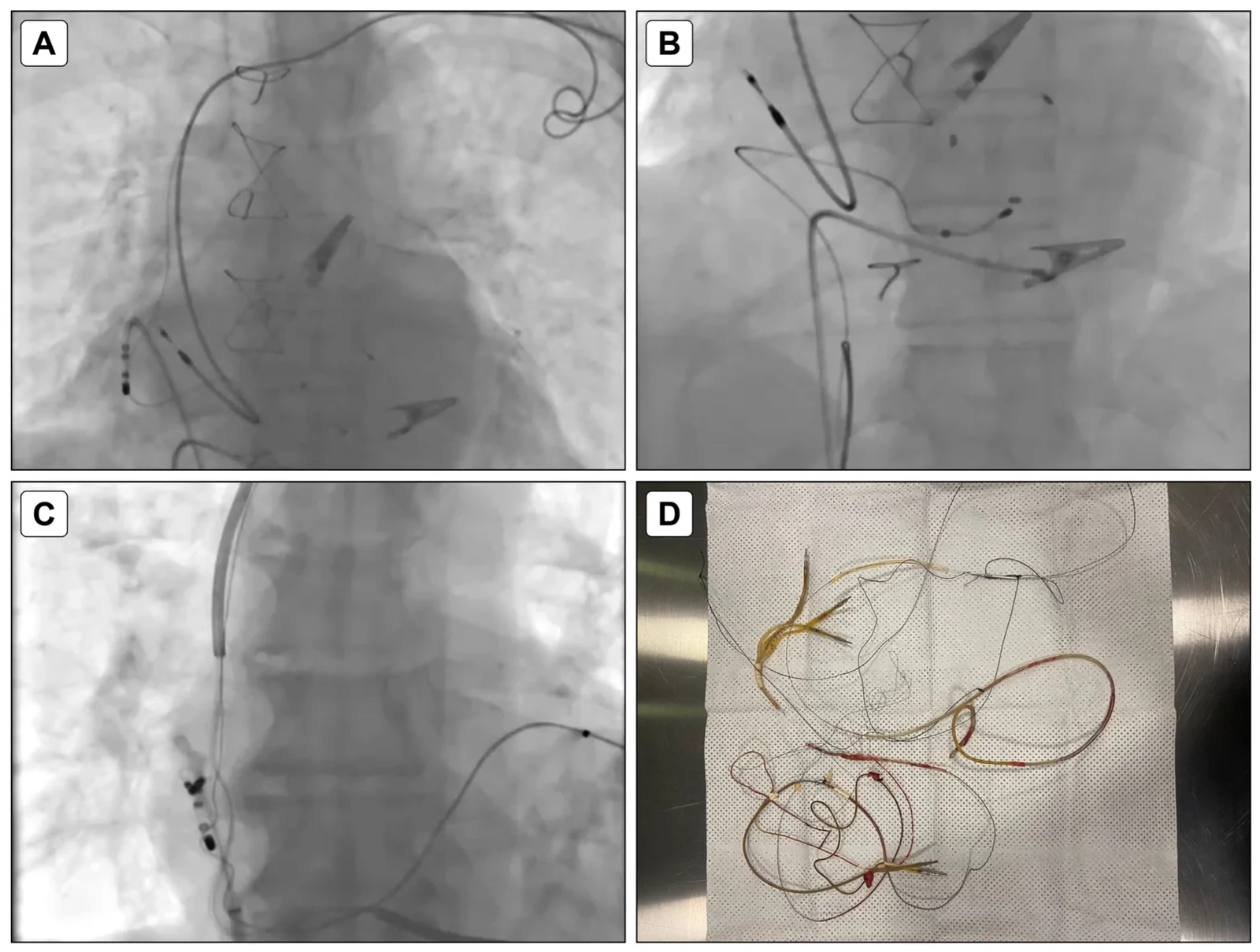
Representative use of the wrap-around rescue technique during transvenous lead extraction. (**A**) Advancement of the RF Marinr steerable ablation catheter toward the target lead through the femoral approach. (**B**) Capture and stabilization of the lead with a 15 mm Amplatz Goose Neck snare during the same procedure shown in panel (**A**). (**C**) In a second patient, the RF Marinr catheter is shown looped around the target lead before snare-assisted traction. (**D**) The completely extracted lead system from the procedure shown in panel (**C**).

Figure 2 summarizes the case-verified pathway. A capturable free end was present in 15/19 bailout procedures. Direct Goose Neck capture alone provided adequate control in two. Thirteen proceeded to wrap-around: capture failed in four, and capture was achieved but stable inferior control remained inadequate in nine. The remaining four procedures had no capturable free end and therefore proceeded directly to RF Marinr wrap-around; three subsequently used the Goose Neck, and one was completed with wrap-around alone. The final femoral retrieval pathway was used in all 19 bailout procedures, with complete removal in 18/19 and clinical procedural success in 19/19. All final bailout retrievals were performed with inferior traction, without concurrent advancement of the superior sheath. Representative procedural images of the wrap-around maneuver are shown in Figure 3.

Supplementary Table S1A presents a case-level comparison of all 19 bailout procedures, including the target lead, dwell time, preceding superior tool, reason for femoral escalation, verified femoral sequence, sheath diameter, complexity findings, and outcome. Supplementary Table S1B summarizes the case-verified decision framework. Supplementary Tables S2–S4 report the annual distribution of the analytic cohort, era descriptions, and safety outcomes; Table S5 summarizes variable availability; Table S5A reports procedural resources; Table S5B reports reimplantation pathways and length of stay; Tables S6 and S7 provide penalized-regression sensitivity analyses and diagnostics; and Supplementary Figure S1 displays extraction-phase resource use by procedural pathway.

After pocket closure, a pressure dressing was applied. It was removed on postprocedural day 1; thereafter, a smaller dressing was applied, and the wound was inspected during subsequent observation.

Post-extraction management was tailored to indication and clinical stability. Same-session permanent reimplantation was performed in 47/78 patients, all with noninfectious indications. None of the 30 infection-related extractions underwent same-session permanent reimplantation. Permanent reimplantation was delayed until culture review and completion of prescribed antimicrobial treatment; exact patient-level reimplantation dates were not retained. Four pacemaker-dependent patients with pocket infection received temporary pacing support. After negative culture results, these four patients underwent permanent-device reimplantation in the right pectoral region, contralateral to the infected pocket. One additional noninfection patient with venous obstruction/superior vena cava syndrome had no same-session reimplantation. Computed-tomography venography was performed; pocket infection, bacteremia, and endocarditis were excluded, and a permanent device was implanted in a subsequent session; the exact date, side, and device type were not retained. Four of eight pacemaker patients with pocket infection were pacemaker dependent and were bridged with temporary pacing until definitive reimplantation. All 48 noninfection patients had a recorded length of stay of one day; the available temporal resolution did not reliably distinguish same-day discharge from overnight observation. Pocket-infection and endocarditis care followed individualized microbiological and antimicrobial assessment. Length of stay was available from hospital records for all 78 patients and is reported in Results. Exact delayed reimplantation date, side, final device type, antimicrobial agent, patient-level treatment duration, and culture-clearance date were not retained. This approach was interpreted in the context of contemporary lead-management guidance and discharge evidence [1].

The extraction date was the time origin for procedural safety and 30-day outcome ascertainment; device surveillance after implantation or reimplantation was anchored separately to the implantation date. The institutional follow-up schedule comprised assessment before discharge, face-to-face wound and pocket review at 48–72 h, repeat wound review at 7–10 days, and device interrogation at approximately 4 weeks. The 30-day device assessment included wound and pocket status, battery condition, P- and R-wave amplitudes, pacing thresholds, lead impedances, pacing percentages, stored arrhythmia episodes, ICD VT/VF zones and delivered therapies, biventricular pacing percentage in CRT recipients, and programming optimization when required. Endocarditis patients who remained hospitalized beyond 30 days underwent the same assessment in hospital and again before discharge when applicable.

### 2.4. Procedural Outcome Definitions

Procedural outcomes were assessed at the patient and lead levels using consensus terminology [1,14]. Complete procedural success required removal of all targeted leads and all associated lead material from the vascular space, without permanently disabling complication or procedure-related death. Lead-level complete removal required no retained material from the individual targeted lead. Clinical success can include a residual lead portion < 4 cm when it does not compromise the procedural goal [1]. The sole incomplete-removal procedure retained a 20 mm distal tip segment of a coronary-sinus (left-ventricular) lead fixed in a distal coronary-sinus branch. It remained unchanged on procedural fluoroscopy and on chest radiography and fluoroscopy at day 30, without migration, embolization, reintervention, or clinically meaningful deterioration in routinely monitored infection-related laboratory parameters. Because the remnant was <4 cm and did not compromise the clinical goal, the procedure was classified as a clinical procedural success while remaining a complete-removal failure. The stricter complete-removal endpoint remained primary.

Bailout escalation was defined as right femoral gooseneck snaring and/or wrap-around after the preceding superior pathway was insufficient. Procedures completed without either bailout technique were classified as primary-only. Bailout procedures were summarized using mutually exclusive procedure-level combinations: snare without wrap-around, snare plus wrap-around, and wrap-around without snare. Any-wrap and any-bailout totals were also reported. Wrap-around pathway salvage was defined as complete procedural success among procedures in which wrap-around was used and was not attributed solely to that maneuver.

### 2.5. Safety Definitions and 30-Day Follow-Up Procedures

Safety outcomes were classified according to consensus definitions [1,13,14] by timing and severity. Intraprocedural events were those occurring or becoming evident from entry into the procedure room until departure, including events related to patient preparation, anesthesia, and wound closure. Early postprocedural events were those occurring or becoming evident after departure from the procedure room through day 30. Events after day 30 and during the first year were considered late postprocedural events but were outside the primary 30-day endpoint. Major complications were life-threatening or fatal events, caused persistent or significant disability, required or prolonged hospitalization, or required major surgical or endovascular intervention. Minor complications required medical treatment or a minor intervention without threatening life or causing persistent or significant functional impairment.

The major-event category included superior vena cava, innominate/subclavian venous, or right-atrial laceration; cardiac avulsion or perforation; tamponade or pericardial effusion requiring intervention; hemothorax requiring intervention; emergency sternotomy, thoracotomy, or endovascular repair; cardiac or respiratory arrest; stroke; massive pulmonary embolism or thromboembolism requiring intervention; severe tricuspid-valve injury requiring intervention; and other permanent consequences. The minor-event category included pericardial effusion not requiring intervention, pocket hematoma or wound complication, non-major bleeding requiring transfusion, venous thrombosis, lead-fragment migration without sequelae, access-site bleeding or arteriovenous fistula, pneumothorax, and new or worsening tricuspid regurgitation not requiring major intervention.

The prespecified 30-day outcomes included all-cause and procedure-related mortality; cardiovascular and noncardiovascular/infection-related mortality; major and minor complications; emergency surgical or endovascular intervention; rehospitalization; repeat intervention or surgery; and migration or subsequent removal of retained lead material. Reimplantation pathway, temporary pacing, wound findings, and length of stay were also assessed. In infection cases, persistent or recurrent bacteremia and recurrent pocket/systemic CIED infection were evaluated through day 30. Outcome ascertainment was based on procedure-room, inpatient, outpatient-clinic, and hospital admission/readmission records. Post-discharge ascertainment before day 30 applied to 74 patients; four endocarditis patients remained hospitalized through day 30. Minimal subcutaneous pocket/incisional hematomas that required neither medical treatment nor a minor procedure were recorded separately as observation-only wound findings rather than minor complications. Complete and clinical procedural success were adjudicated using consensus definitions, and lead-level complete removal was reported separately.

### 2.6. Statistical Analysis

Each patient was treated as an independent observation. Continuous variables were summarized as mean with standard deviation (SD) or median with interquartile range (IQR), as appropriate, and categorical variables as counts and percentages. Age and left ventricular ejection fraction were compared using Welch’s *t* test; skewed continuous variables using the Mann–Whitney U test; and categorical variables using Fisher’s exact test or the chi-square test, as appropriate. All tests were two-sided, with no multiplicity adjustment for exploratory comparisons. Extraction-phase duration was defined from skin incision/pocket opening to removal of the final target lead; total skin-to-skin duration extended to final wound closure; and the post-extraction interval was total duration minus extraction-phase duration. These duration endpoints were available for all 78 procedures. Fluoroscopy time and system-reported air kerma were limited to the extraction phase and excluded same-session implantation. Primary-only and bailout resource metrics were compared using exploratory Mann–Whitney U tests. Total-case fluoroscopy was not analyzed because it could not be reconciled reliably with the extraction-phase records, and directly measured total-case air kerma was unavailable. No missing clinical or lead-design variable was imputed. For zero 30-day events, a two-sided exact Clopper–Pearson 95% upper confidence limit was reported.

The conventional unadjusted model examined the association between target-lead dwell time and any bailout use (snare and/or wrap-around) using maximum-likelihood logistic regression. The primary exploratory adjusted analysis used Firth penalized logistic regression with three clinically selected predictors specified before model fitting: target-lead dwell time in years, calendar era (2020–2025 versus 2014–2019), and number of targeted leads. With 19 bailout events, this three-predictor model remained deliberately parsimonious and exploratory. Odds ratios were accompanied by profile penalized-likelihood 95% confidence intervals and penalized likelihood-ratio *p* values; no automated variable selection was used.

A sensitivity Firth model replaced targeted-lead count with a binary marker for any targeted ICD or coronary-sinus lead; this was a replacement predictor, not an additional fourth covariate. Linearity was examined by adding a centered quadratic dwell-time term to the adjusted Firth model. Influence was assessed with model leverage and 78 leave-one-out Firth fits. Procedural pathways and key outcome proportions were summarized with Wilson 95% confidence intervals. Clinical procedural success was adjudicated using the consensus < 4 cm retained-fragment criterion together with the documented absence of adverse impact [1,14].

In the exploratory lead-type analysis, patients contributed to each attempted lead category present, so groups were not mutually exclusive. Bailout, snare, and wrap-around use were attributed to the recorded bailout target, with patients as the denominator for pathway use and leads as the denominator for lead-level removal. Verified target-lead dwell was available for all 78 patients. Detailed fixation mechanism, manufacturer/model, ICD coil configuration, venous anatomy, calcification, abandoned-lead status, prior lead intervention, and lead-fracture status were not consistently recoverable because all index implants had been performed outside the study center; these variables were not imputed or entered in multivariable analyses.

**Figure 4:**
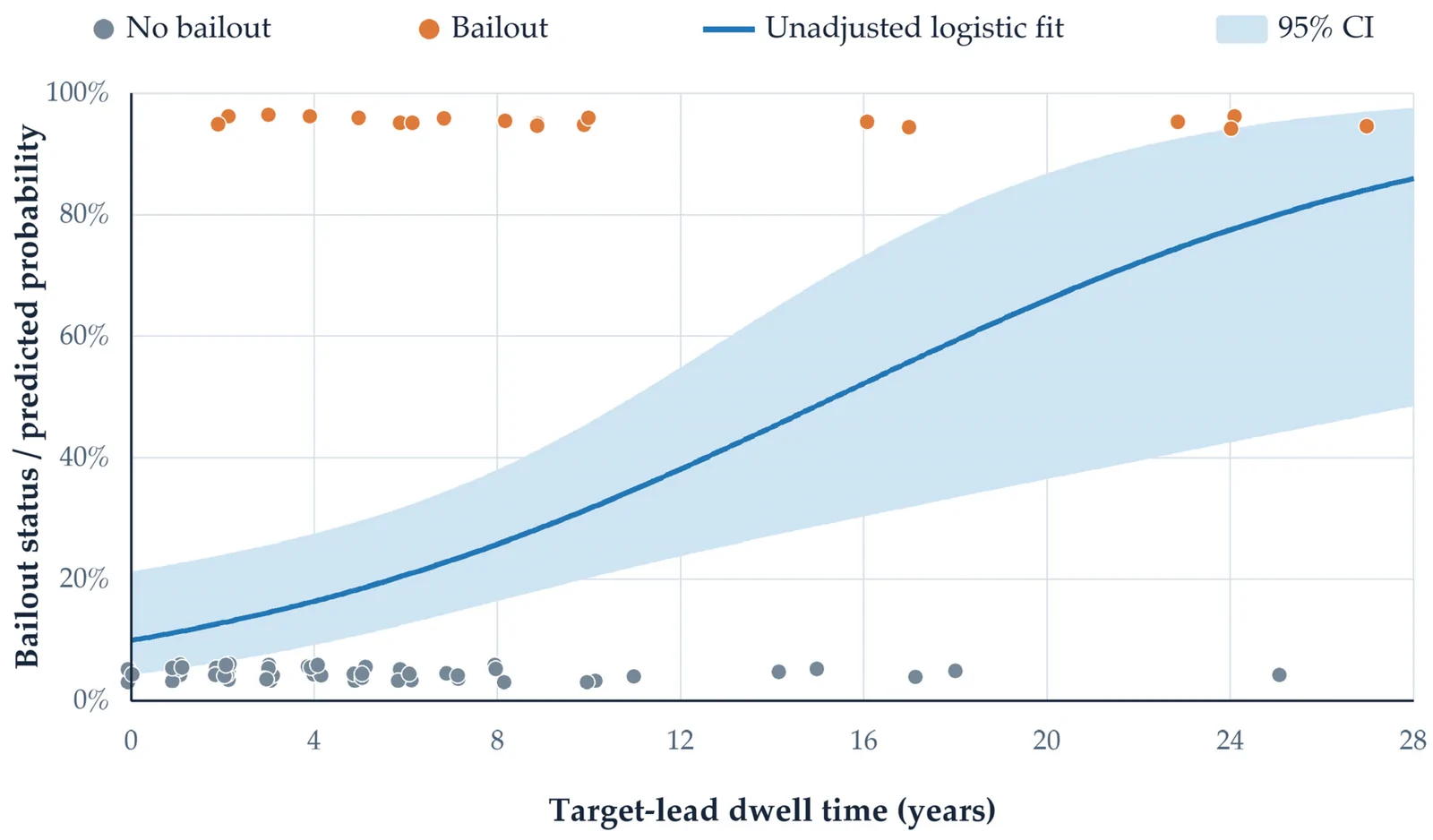
Target-lead dwell time and bailout use. Points show individual patients with deterministic horizontal and vertical jitter to improve visibility; the solid line is the model-derived probability from univariable logistic regression, and the shaded band is the 95% confidence interval. Note: Jitter has no analytical meaning. Target-lead dwell time was calculated as extraction year minus target-lead implantation year; a value of 0 indicates implantation and extraction within the same calendar year. The curve is unadjusted and should not be interpreted as a causal or clinical decision threshold.

Data management and statistical analyses were performed in Python 3.12.13 using pandas 2.2.3 and NumPy 2.3.5. A custom Python implementation of Firth penalized logistic regression maximized the Jeffreys-prior penalized likelihood; modified-score calculations were checked against a finite-difference gradient, and convergence, leverage, penalized likelihood-ratio tests, and leave-one-out fits were examined. Figure 1 and Figure 2 were generated deterministically using Pillow 12.3.0; the procedural flowchart in Figure 2 was generated from an author-approved decision structure. Figure 3 comprises representative source procedural images without superimposed annotations. Figure 4 and Supplementary Figure S1 were generated deterministically using Pillow 12.3.0 and were based directly on the de-identified analysis data.

### 2.7. Use of Generative Artificial Intelligence

Generative AI-assisted tools were used solely to assist English-language refinement, consistency checking, and table and document formatting. They were not used to generate or alter patient-level data, determine eligibility, define or adjudicate clinical outcomes, replace investigator-led statistical analysis, or generate scientific data graphics. All data plots and the procedural flowchart were produced deterministically from author-approved code and data structures. All outputs were reviewed, verified, and edited by the authors.

## 3. Results

### 3.1. Baseline and Procedural Characteristics

The final analytic cohort included 78 patients with a mean age of 63.41 years (SD, 14.09); 55 (70.5%) were men. Mean left ventricular ejection fraction was 42.79% (SD, 13.67). Heart failure and coronary artery disease were present in 52 (66.7%) and 45 patients (57.7%), respectively.

Median target-lead dwell time was 5.0 years; the interquartile range (IQR) was 2.0–8.0 years, and the observed range was 0–27 years. Four patients had a value of 0 years because implantation and extraction occurred within the same calendar year. Patients requiring bailout had a median target-lead dwell time of 9.0 years (IQR, 5.5–16.5), compared with 4.0 years (IQR, 2.0–6.0) in the primary-only group (*p* < 0.001). Among the 17 procedures using wrap-around, median target-lead dwell time was 8.0 years (IQR, 5.0–16.0; range, 2–27). Other baseline comparisons were not statistically significant (Table 1). The early era included 45 procedures (7.5 included procedures/year) and the contemporary era 33 (5.5/year). Annual included-cohort counts ranged from 1 to 17 and varied without a consistent monotonic pattern. Bailout use was 11/45 (24.4%) versus 8/33 (24.2%), wrap-around use 10/45 (22.2%) versus 7/33 (21.2%), and complete procedural success 45/45 (100.0%) versus 32/33 (97.0%), respectively. Annual included-cohort counts are shown in Supplementary Table S2 and era summaries in Supplementary Table S3. These included-cohort counts are not complete center-wide annual volume and should not be interpreted as a program volume or learning trend. This was a small, single-operator program performing approximately 10 TLE procedures per year. The first recorded wrap-around case occurred in 2015. Regarding extraction-sheath availability, the Evolution^®^ Mechanical Dilator Sheath Set was used in 2014 and the Evolution^®^ RL Controlled-Rotation Dilator Sheath Set from 2015 onward [15]; historical changes in the escalation threshold were not systematically documented.

Duration data were complete for all 78 procedures. Median extraction-phase duration was 79.7 min (IQR, 58.3–111.4; range, 24–216), and median total skin-to-skin duration was 110.1 min (IQR, 82.6–148.7; range, 39–271). Extraction-phase duration was 71.0 min (IQR, 58.0–106.5) in the primary-only group and 93.8 min (IQR, 77.5–119.8) in the bailout group (exploratory *p* = 0.249); total skin-to-skin duration was 108.4 min (IQR, 80.0–134.7) and 126.0 min (IQR, 100.4–155.1), respectively (*p* = 0.222). The overall post-extraction interval was 32.5 min (IQR, 17.1–39.7; range, 10–64.9). It was 38.4 min (IQR, 33.5–43.6) with same-session reimplantation and 16.0 min (IQR, 14.5–17.8) without same-session reimplantation. Median extraction fluoroscopy time was 21.8 min (IQR, 6.9–35.5; range, 2.0–76.0), and median fluoroscopy-system-reported air kerma was 324.5 mGy (IQR, 125.6–1036.8; range, 55.0–3624.0). Bailout procedures had longer fluoroscopy than primary-only procedures (31.4 vs. 16.6 min; *p* = 0.011), whereas air kerma did not differ (282.2 vs. 346.4 mGy; *p* = 0.736) (Supplementary Table S5A and Supplementary Figure S1).

### 3.2. Indications, Device Types, and Attempted-Lead Characteristics

Lead dysfunction was the most common indication for extraction (36/78, 46.2%). Infection-related indications accounted for 30/78 procedures (38.5%): CIED pocket infection in 26/78 (33.3%) and endocarditis in 4/78 (5.1%). Same-session reimplantation was performed in 47/78 patients (60.3%), all in the noninfection group; none of the 30 infection-related cases received a permanent device during the extraction session. The mutually exclusive same-session indication categories were lead dysfunction without a CRT-upgrade code in 36/47, lead dysfunction plus CRT upgrade in 2/47, CRT-D upgrade without a lead-dysfunction code in 4/47, lead-related tricuspid valve dysfunction in 3/47, lead repositioning in 1/47, and pericardial penetration in 1/47. Thus, six same-session procedures carried a CRT-upgrade indication. The recorded baseline-device entry described the system present at the extraction encounter and was not interpreted as the newly implanted device type. All infection-related cases followed the delayed reimplantation pathway after microbiological review and prescribed antimicrobial therapy. Four of eight pacemaker patients with pocket infection required temporary pacing as a bridge. CRT-D and dual-chamber pacemakers were the most frequent baseline device types. The recorded principal superior extraction method was manual in half of the procedures; remaining cases involved Cook, Spectranetics/Philips, or Bulldog equipment (Table 2).

The 78 patients had 175 transvenous leads in situ. Extraction was attempted for 156 leads; 19 functioning leads without an extraction indication were retained. Attempted leads included 59 atrial, 25 right-ventricular pacing, 22 coronary-sinus (left-ventricular), and 50 right-ventricular ICD leads (Table 3).

### 3.3. Complete Procedural Success and Bailout Pathways

Complete procedural success was observed in 77 of 78 patients (98.7%; 95% CI, 93.1–99.8), and lead-level complete removal in 155 of 156 targeted leads (99.4%; 95% CI, 96.5–99.9). Clinical procedural success was 78/78 (100.0%; 95% CI, 95.3–100.0) because the only retained material was a 20 mm distal coronary-sinus lead tip that remained fixed in a distal coronary-sinus branch without migration, embolization, or reintervention through day 30. The primary-only pathway was sufficient and successful in all 59 patients. The 19 bailout procedures comprised 16 using both Goose Neck and wrap-around, two using Goose Neck without wrap-around, and one using wrap-around without Goose Neck. Bailout achieved complete removal in 18/19, including 16/17 procedures using wrap-around (Table 4). Six bailout procedures had a CIED pocket-infection indication and 13 were noninfectious; none was extracted for endocarditis. In the six pocket-infection procedures, blood cultures were obtained because systemic involvement was suspected, but no vegetation was documented, and the extraction indication remained pocket infection. Venous occlusion was documented in one procedure, abandoned leads in three, and a previous unsuccessful extraction in one; none of the 19 patients had prior sternotomy.

### 3.4. Lead Dwell Time and Bailout Use

In the conventional unadjusted model, each 1-year increase in target-lead dwell time was associated with higher bailout odds (OR, 1.15; 95% CI, 1.06–1.26; *p* = 0.001). In the primary exploratory Firth model, the association persisted after adjustment for era and number of targeted leads (adjusted OR per year, 1.14; 95% CI, 1.05–1.25; *p* < 0.001). Estimates for the contemporary era (adjusted OR, 1.12; 95% CI, 0.36–3.48; *p* = 0.848) and each additional targeted lead (adjusted OR, 1.48; 95% CI, 0.72–3.14; *p* = 0.283) were imprecise. In the sensitivity model using ICD/coronary-sinus involvement, the target-lead dwell-time estimate was similar (adjusted OR, 1.16; 95% CI, 1.07–1.29; *p* < 0.001). The adjusted quadratic dwell term did not improve fit (penalized likelihood-ratio *p* = 0.499); primary-model leverage was at most 0.184, and leave-one-out dwell-time ORs ranged from 1.13 to 1.17 (Table 5).

### 3.5. Bailout Use and Outcomes by Attempted Lead Type

The highest crude bailout-target proportions were observed for right-ventricular pacing leads (26.1%) and right-ventricular ICD leads (22.9%), followed by coronary-sinus (left-ventricular) leads (13.6%) and atrial leads (8.8%) (Table 6). These overlapping descriptive data do not provide independent lead-type risk estimates.

### 3.6. Outcomes by Principal Superior Extraction Method

Bailout was used in 20.5% of procedures recorded as manual, 31.8% of those involving Cook equipment, 18.8% of those involving Spectranetics/Philips equipment, and in the single Bulldog Lead Extender case. Complete procedural success was 97.4%, 100%, 100%, and 100%, respectively (Table 7). These categories represent the recorded principal superior method, not complete ordered tool histories.

### 3.7. Periprocedural Safety and 30-Day Clinical Outcomes

Study-center records provided 30-day outcome ascertainment for all 78 patients. No all-cause, procedure-related, cardiovascular, or noncardiovascular/infection-related death; consensus-defined major or minor complication; emergency surgical/endovascular intervention; readmission; or repeat intervention was documented through day 30 (all 0/78). Four patients (5.1%) developed minimal subcutaneous pocket/incisional hematomas: one after same-session reimplantation and three after extraction without same-session reimplantation. All four followed primary-only procedures, were managed by observation alone, and required no medication, transfusion, evacuation, or other intervention; they were therefore reported separately from formal minor complications. The retained 20 mm distal coronary-sinus (left-ventricular) lead tip remained fixed in a distal coronary-sinus branch on day-30 chest radiography and fluoroscopy, with no migration, embolization, or reintervention. The exact two-sided 95% upper confidence limit for a zero-event formal complication rate was 4.6%. Among 30 infection-related cases, no persistent/recurrent bacteremia or recurrent pocket/systemic CIED infection was identified through day 30 (all 0/30). Four pacemaker-dependent pocket-infection patients received temporary pacing and later underwent contralateral right-pectoral permanent-device reimplantation after negative cultures; the remaining infection cases also followed delayed reimplantation after culture review and antimicrobial treatment. The noninfection patient with superior vena cava syndrome underwent computed-tomography venography and subsequent-session implantation after pocket infection, bacteremia, and endocarditis were excluded. Four endocarditis patients remained hospitalized through day 30. Median length of stay was 1 day overall (IQR, 1–3; range, 1–45), 1 day for noninfection (IQR, 1–1), 3 days for pocket infection (IQR, 3–3.75), and 42 days for endocarditis (IQR, 37.5–44.25). Complete procedural success was 77/78 (98.7%) and clinical procedural success was 78/78 (100.0%).

## 4. Discussion

In this single-operator analytic cohort, complete removal was observed in nearly all patients, including 16 of 17 selected procedures in which wrap-around was used. Case-level review clarified the mechanics: two procedures were completed by direct Goose Neck capture alone; 16 used Goose Neck with RF Marinr wrap-around and subsequent lead-segment recapture; and one used RF Marinr wrap-around without a snare. All bailout retrievals were completed through the right femoral route. These observations improve the transparency and procedural specificity of the pathway description but remain descriptive pathway-level data; the independent effect of wrap-around cannot be isolated from snaring, preceding tools, or case selection.

Lead dysfunction and pocket infection were the leading indications, consistent with earlier single-center reports [3,10,16]. The observed complete-removal rate was comparable to ELECTRa, PROMET, and LExICon [2,17,18], but such cross-study comparisons cannot account for case selection, lead burden, tool availability, or outcome definitions. The contemporary SAFeTY TLE analysis likewise illustrates that outcome interpretation and preprocedural risk assessment require a multivariable clinical context rather than attribution to a single maneuver [19].

The ELECTRa Registry Outcome Score (EROS) further demonstrates that TLE risk stratification should integrate multiple clinical and procedural features rather than lead dwell time alone [20]. Mdaihly et al. analyzed a prospectively maintained, 4989-patient high-volume-center registry and identified 163 procedures (3.3%) requiring a femoral bailout; adding the femoral workstation increased cumulative clinical success from 96.4% to 99.1% and complete procedural success from 94.6% to 97.3%, without a statistically significant increase in major complications [21]. In contrast, the present report describes a small, single-operator program performing approximately 10 TLE procedures per year. It is enriched for selected wrap-around cases and cannot estimate a comparative treatment effect.

Longer target-lead dwell time was associated with bailout use in both the unadjusted model and the parsimonious Firth model adjusting for calendar era and targeted-lead count. The association was similar when ICD/coronary-sinus involvement replaced lead count. The dwell-time OR remained 1.13–1.17 across 78 leave-one-out refits, and the quadratic-term test was not significant (*p* = 0.499). Target-lead dwell time was calculated separately for each extracted lead from its actual implantation year. In bailout procedures, the analysis used the lead that prompted femoral escalation; when two leads had been implanted simultaneously, both had the same implantation year and therefore the same dwell time. Integer calendar-year resolution does not capture exact elapsed days. The estimate is exploratory, noncausal, and not a clinical threshold.

Lead-type findings were exploratory because patients could contribute to more than one lead category. The highest crude bailout-target proportions were observed for right-ventricular pacing and ICD leads. These overlapping descriptive data do not provide independent lead-type risk estimates; lead type should therefore be interpreted together with dwell time and the number of targeted leads. Reports addressing abandoned leads, intraprocedural lead breakage, ICD coil/fixation characteristics, and ICD lead dwell beyond 10 years further support multidimensional assessment of extraction complexity [22,23,24,25]. These characteristics were not recoverable case by case in the present cohort and were not imputed.

Bailout-target complete removal was observed for every atrial, right-ventricular pacing, and right-ventricular ICD target. Two of three coronary-sinus (left-ventricular) targets were completely removed; the only incomplete case retained a 20 mm distal tip segment of the coronary-sinus lead. This remained a complete-removal failure, but the <4 cm remnant and absence of clinical compromise met the consensus definition of clinical procedural success [1,14]. The tip was fixed in a distal coronary-sinus branch and remained unchanged on procedural and day-30 imaging without migration, embolization, or reintervention. One event cannot support a lead-type risk inference; in a 182-case coronary-sinus series, an apparent difference by lead design disappeared after adjustment for lead age [26].

A liberal combined superior-femoral approach has previously been evaluated as a systematic access strategy [27]. The 2025 network meta-analysis included 11 nonrandomized studies and one randomized trial and found no statistically significant difference among techniques for patient-level clinical or lead-level procedural success, despite treatment rankings and a lower significant-complication estimate for femoral versus laser approaches [28]. Its study-level comparisons do not isolate wrap-around rescue. The updated 2026 consensus supports familiarity with both superior and femoral approaches and access to rescue expertise but does not identify one bailout maneuver as superior [1]. Together with the Mdaihly registry, these benchmarks support procedural flexibility while limiting causal claims from our descriptive series.

The present wrap-around maneuver belongs to an established family of femoral and combined-access techniques. Jo et al. described an ablation-catheter/gooseneck-snare closed loop; Uslu et al. reported femoral gooseneck snaring; Yap et al. evaluated a liberal combined superior-femoral approach; tandem reports used femoral control or countertraction to stabilize the lead; and later case/series reports described steerable wire loops, ablation-catheter/snare loops, and wrapping for fractured leads [9,12,27,29,30,31,32,33]. These reports differ in access strategy, target condition, loop construction, and whether femoral tools were primary, adjunctive, or bailout. Our contribution is the description of wrap-around within a case-verified stepwise bailout pathway, without a claim of technical novelty or superiority.

Study-center records provided 30-day outcome ascertainment for 78/78 patients. No death, consensus-defined major/minor complication, readmission, or reintervention was documented; four minimal subcutaneous hematomas required observation alone and are reported separately. The exact 95% upper bound of 4.6% for a zero-event formal complication rate and the small cohort preclude exclusion of uncommon harm. This *n*/*N* reporting and minimum 30-day window follow consensus and large-registry conventions, while the absence of events should not be equated with comparative safety. Gianni et al. found selected same-day discharge feasible after uncomplicated TLE. All 48 noninfection patients in our cohort had a recorded one-day length of stay, but the available temporal resolution did not reliably distinguish same-day discharge from overnight observation; pocket-infection and endocarditis groups had longer observed lengths of stay [2,14,34,35].

Extraction decisions also belong within lifetime CIED management. The 2026 consensus emphasizes considering patient longevity, future vascular access, cumulative lead burden, and consequences of later device complications whenever a system is implanted or revised [1]. In selected symptomatic Brugada syndrome populations, a small 2025 meta-analysis of three controlled studies associated substrate ablation with lower ventricular-fibrillation recurrence than standard ICD-based therapy, while emphasizing the limited evidence base [36]. Conduction-system pacing may reduce dyssynchrony in selected heart-failure patients but introduces evolving lead-management considerations and still lacks definitive long-term randomized evidence [37]. These sources provide lifetime-device context and do not evaluate wrap-around extraction efficacy.

### Limitations

This retrospective, single-center, single-operator study reflects a small private-hospital program and is susceptible to referral, operator, and center effects. Following an institutional archive transition, complete records were retrievable for 82 procedures; exclusion of four image-only records may have introduced selection bias. The 12-year study period also spanned changes in experience and equipment that era adjustment cannot fully resolve.

This retrospective, single-center, single-operator study included only 19 bailout procedures, limiting comparative, multivariable, and safety inference. Historical lead model, fixation mechanism, ICD coil configuration, and systematic calcification grading could not be recovered consistently because the original implants were performed elsewhere; these omissions precluded a validated complexity score. Directly measured total-case air kerma was unavailable, total-case fluoroscopy was not sufficiently reconciled for analysis, and study-center records may have missed care delivered elsewhere. These limitations preclude causal claims about the independent efficacy or comparative safety of wrap-around.

## 5. Conclusions

In this retrospective single-center cohort, a case-verified stepwise femoral bailout pathway achieved complete removal in 18/19 bailout procedures and in 16/17 procedures using wrap-around. The pathway comprised two direct-Goose-Neck-only procedures, 16 combined Goose Neck/RF Marinr procedures, and one wrap-around-only procedure; all final bailout retrievals were femoral. Clinical procedural success was achieved in 78/78 procedures and complete procedural success in 77/78. No consensus-defined major or minor complication was documented through day 30, while four minimal subcutaneous hematomas required observation only. Bailout procedures had longer extraction fluoroscopy in an exploratory comparison, and longer target-lead dwell remained associated with bailout in a small exploratory Firth model. The results support the feasibility of this structured bailout pathway in the present cohort and provide a detailed framework for future evaluation; they do not establish external reproducibility, comparative efficacy, or comparative safety.

## Figures and Tables

**Table 1:** Baseline characteristics overall and by extraction pathway.

Characteristic	Overall (*n* = 78)	Primary Only (*n* = 59)	Bailout (*n* = 19)	*p* Value
Age, years	Mean: 63.41; SD: 14.09	Mean: 64.54; SD: 13.26	Mean: 59.89; SD: 16.29	0.213
Male sex, *n* (%)	55 (70.5)	41 (69.5)	14 (73.7)	0.727
NYHA class III-IV, *n* (%)	13 (16.7)	9 (15.3)	4 (21.1)	0.724
LVEF, %	Mean: 42.79; SD: 13.67	Mean: 42.12; SD: 13.26	Mean: 44.89; SD: 15.04	0.445
End-stage renal disease, *n* (%)	4 (5.1)	3 (5.1)	1 (5.3)	1.000
Atrial fibrillation, *n* (%)	20 (25.6)	15 (25.4)	5 (26.3)	1.000
Hypertension, *n* (%)	40 (51.3)	31 (52.5)	9 (47.4)	0.695
Diabetes mellitus, *n* (%)	13 (16.7)	12 (20.3)	1 (5.3)	0.169
Heart failure, *n* (%)	52 (66.7)	41 (69.5)	11 (57.9)	0.351
Coronary artery disease, *n* (%)	45 (57.7)	35 (59.3)	10 (52.6)	0.608
Target-lead dwell time, years	Median: 5.0; IQR: 2.0–8.0	Median: 4.0; IQR: 2.0–6.0	Median: 9.0; IQR: 5.5–16.5	<0.001
Implanted leads per patient	Median: 2.0; IQR: 2.0–3.0	Median: 2.0; IQR: 2.0–3.0	Median: 2.0; IQR: 2.0–3.0	0.383
Attempted leads per patient	Median: 2.0; IQR: 1.0–2.0	Median: 2.0; IQR: 1.0–2.0	Median: 2.0; IQR: 2.0–2.5	0.276
Target-lead dwell time > 10 years, *n* (%)	12 (15.4)	6 (10.2)	6 (31.6)	0.061

Note: *p*-values compare the primary-only and bailout pathways using the tests described in Statistical Analysis. IQR, interquartile range; LVEF, left ventricular ejection fraction; NYHA, New York Heart Association; SD, standard deviation.

**Table 2:** Clinical indications, device types, and principal superior extraction-method categories.

Category	Subgroup	*n* (%)
Indication	Lead dysfunction	36 (46.2)
	CIED pocket infection	26 (33.3)
	Endocarditis	4 (5.1)
	CRT-D upgrade	4 (5.1)
	Lead dysfunction with CRT upgrade	2 (2.6)
	Lead-related tricuspid valve dysfunction	3 (3.8)
	Electrode reposition	1 (1.3)
	Superior vena cava syndrome	1 (1.3)
	Pericardial penetration	1 (1.3)
Device	CRT-D	26 (33.3)
	Dual-chamber pacemaker	24 (30.8)
	Dual-chamber ICD	17 (21.8)
	Single-chamber ICD	8 (10.3)
	Single-chamber pacemaker	2 (2.6)
	Atrial pacemaker	1 (1.3)
Principal superior method	Manual	39 (50.0)
	Cook equipment	22 (28.2)
	Spectranetics/Philips equipment	16 (20.5)
	Bulldog Lead Extender	1 (1.3)

Note: The method category represents the principal superior extraction method recorded, rather than every tool used. Cook equipment may include Cook lead-control devices and/or the mechanical dilator sheath available in that period: Evolution^®^ in 2014 and Evolution^®^ RL from 2015 onward. CRT-D, cardiac resynchronization therapy defibrillator; ICD, implantable cardioverter-defibrillator. Indication categories were mutually exclusive; CIED pocket infection and endocarditis accounted for 26/78 and 4/78 procedures, respectively.

**Table 3:** Implanted- and attempted-lead characteristics.

Category	Subgroup	*n*/*N* (%)
Implanted leads per patient	1 lead	11/78 (14.1)
	2 leads	39/78 (50.0)
	3 leads	26/78 (33.3)
	4 leads	2/78 (2.6)
Leads attempted per patient	1 lead	21/78 (26.9)
	2 leads	38/78 (48.7)
	3 leads	17/78 (21.8)
	4 leads	2/78 (2.6)
Attempted lead type	Atrial	59/156 (37.8)
	Right-ventricular pacing	25/156 (16.0)
	Coronary-sinus (left-ventricular)	22/156 (14.1)
	Right-ventricular ICD	50/156 (32.1)

**Table 4:** Mutually exclusive bailout technique combinations and complete procedural success.

Pathway	Patients, *n* (% of Cohort)	Final Complete Procedural Success
No bailout required	59 (75.6)	59/59 (100.0)
Primary approach insufficient, requiring bailout	19/78 (24.4)	18/19 (94.7)
Snare without wrap-around	2 (2.6)	2/2 (100.0)
Wrap-around plus snare	16 (20.5)	15/16 (93.8)
Wrap-around without snare	1 (1.3)	1/1 (100.0)
Any wrap-around	17 (21.8)	16/17 (94.1)
Any bailout	19 (24.4)	18/19 (94.7)

**Table 5:** Logistic-regression estimates for any bailout use.

Model	Covariate	OR (95% CI)	*p*
Unadjusted maximum likelihood	Target-lead dwell time, per year	1.15 (1.06–1.26)	0.001
Primary Firth	Target-lead dwell time, per year	1.14 (1.05–1.25)	<0.001
Primary Firth	Contemporary era (2020–2025 vs. 2014–2019)	1.12 (0.36–3.48)	0.848
Primary Firth	Number of targeted leads, per additional lead	1.48 (0.72–3.14)	0.283

Note: The Firth model used profile penalized-likelihood confidence intervals and penalized likelihood-ratio *p* values. It included 19 bailout events and three clinically selected predictors specified before model fitting. Target-lead dwell time was calculated as extraction year minus target-lead implantation year. CI, confidence interval; OR, odds ratio.

**Table 6:** Bailout use and outcomes by attempted lead type.

Lead Type	Patients with Attempted Type, *n*	Leads, *n*	Bailout Target, *n* (%)	Bailout-Target Complete Removal, *n*/*N* (%)	Snare, *n* (%)	Wrap-Around, *n* (%)	Lead-Level Complete Removal, *n*/*N* (%)
Atrial	57	59	5 (8.8)	5/5 (100.0)	5 (8.8)	5 (8.8)	59/59 (100.0)
Right-ventricular pacing	23	25	6 (26.1)	6/6 (100.0)	6 (26.1)	5 (21.7)	25/25 (100.0)
Coronary-sinus (left-ventricular)	22	22	3 (13.6)	2/3 (66.7)	3 (13.6)	3 (13.6)	21/22 (95.5)
Right-ventricular ICD	48	50	11 (22.9)	11/11 (100.0)	10 (20.8)	10 (20.8)	50/50 (100.0)

Note: Lead-type groups are not mutually exclusive. Attempted leads and lead-level complete removal use lead counts; bailout, snare, and wrap-around denominators are patients with at least one attempted lead of that type. Bailout tools were attributed to the recorded bailout target. Row-specific counts must not be summed across overlapping lead types. The only incompletely removed target was a coronary-sinus (left-ventricular) lead. No between-group *p*-values were calculated.

**Table 7:** Primary and bailout outcomes by principal superior extraction-method category.

Method Category	Total *n*	Primary Only *n* (%)	Primary-Only Success	Bailout *n* (%)	Bailout Success	Complete Success
Manual	39	31 (79.5)	31/31 (100.0)	8 (20.5)	7/8 (87.5)	38/39 (97.4)
Cook equipment	22	15 (68.2)	15/15 (100.0)	7 (31.8)	7/7 (100.0)	22/22 (100.0)
Spectranetics/Philips	16	13 (81.2)	13/13 (100.0)	3 (18.8)	3/3 (100.0)	16/16 (100.0)
Bulldog Lead Extender	1	0 (0.0)	Not applicable	1 (100.0)	1/1 (100.0)	1/1 (100.0)

Note: Categories describe the principal superior extraction method and do not exclude subsequent femoral bailout. The Bulldog category contains one patient. Results are descriptive only; no between-category hypothesis testing or comparative efficacy inference was performed.

## Data Availability

The data presented in this study are available from the corresponding author upon reasonable request and subject to institutional and ethical approval. The data are not publicly available because they contain potentially identifiable clinical information and are subject to patient-confidentiality restrictions.

## Supplementary Materials

The following supporting information can be downloaded at: https://www.mdpi.com/article/10.3390/jcm15176832/s1. Supplementary Methods and Data Interpretation; Table S1A, case-level comparison of all bailout procedures; Table S1B, case-verified procedural decision framework; Table S2, annual distribution of the analytic cohort and center-volume context; Table S3, era description; Table S4, periprocedural and 30-day outcomes; Table S5, variable availability; Table S5A, procedural duration, fluoroscopy, and air kerma; Table S5B, reimplantation pathway and length of stay; Tables S6 and S7, Firth models and diagnostics; Figure S1, extraction resource use by procedural pathway.
