# Supplementary material for: Transvenous Lead Extraction Using a Stepwise Bailout Strategy: A Single-Center Experience with Wrap-Around Rescue in Selected Bailout Cases
Source: J Clin Med. 2026 Sep 3;15(17):6832. doi: 10.3390/jcm15176832 (PMC13566201; doi:10.3390/jcm15176832)
Supplement: Supplementary file 1 [file jcm-15-06832-s001.zip › Transvenous_Lead_Extraction_Supplementary_Material_Clean.pdf]

Supplementary Material

Supplementary Material: Wrap-Around Rescue, Temporal Description, Statistical Analyses, and Data Availability

Ebru Şahin [1,*], Hakan Aksoy [1], Cansu Selcan Akdeniz [1], Begüm Yetiş Sayın [1], Sercan Okutucu [2] and Ali Oto [1]

[1] Department of Cardiology, Memorial Ankara Hospital, Çankaya, 06520 Ankara, Türkiye; haksoy63@gmail.com (H.A.); scansuakdeniz@gmail.com (C.S.A.); begumyts@yahoo.com (B.Y.S.); alioto@tksv.org (A.O.)
[2] Department of Cardiology, Lokman Hekim University Ankara Hospital, Sincan 06936 Ankara, Türkiye; sercanokutucu@gmail.com
* Correspondence: ebru_sahin@ymail.com

Associated manuscript: Transvenous Lead Extraction Using a Stepwise Bailout Strategy: A Single-Center Experience with Wrap-Around Rescue in Selected Bailout Cases

1. Supplementary Methods and Data Interpretation

All 19 bailout procedures were included in the case-level review. Case labels B01–B19 are privacy-preserving study labels rather than medical-record numbers and do not reproduce the source-row order. No direct identifiers, exact procedure dates, or demographic combinations are presented. Dwell time was calculated separately for each target lead in integer years as extraction year minus its documented implantation year. In bailout procedures, the analysis used the lead that prompted femoral escalation. When two target leads had been implanted during the same procedure, both had the same implantation year and therefore the same dwell time. A value of 0 denotes implantation and extraction within the same calendar year. Target-lead dwell values were available for all 78 patients.

Procedural reports, material records, and fluoroscopic images were reviewed for all 19 bailout procedures. This review established the reason for femoral escalation, the presence or absence of a capturable free lead end, the ordered Goose Neck and RF Marinr sequence, the right-femoral route, the 10-Fr introducer, target-adapted 9- or 11-Fr dissection-sheath selection, and the final direction of extraction. Procedure-level combinations were 16 Goose Neck plus wrap-around, two Goose Neck without wrap-around, and one wrap-around without Goose Neck.

For clinical-indication context, 26/78 patients (33.3%) had CIED pocket infection and 4/78 (5.1%) had endocarditis; infection-related indications therefore totaled 30/78 (38.5%). Same-session reimplantation was performed in 47/78 patients, all with noninfectious indications. None of the 30 infection cases received same-session permanent reimplantation; all followed a delayed pathway after culture review and prescribed antimicrobial treatment. Four of eight pacemaker patients with pocket infection were bridged with temporary pacing. Length of stay was available for all 78 patients and is summarized in Table S5B.

Extraction-only procedure duration, fluoroscopy time, and fluoroscopy-system-reported air kerma were complete for all 78 patients and were obtained from validated fluoroscopy-system records. Same-session total skin-to-skin duration was analyzed separately in 47 cases. Total-case fluoroscopy failed source-quality-control review and was

excluded; total-case air kerma was not directly measured, and linked/cached estimates were excluded rather than imputed.

2. Results and Interpretation

Across all 19 bailout procedures, 16 used both Goose Neck and wrap-around, two used Goose Neck without wrap-around, and one used wrap-around without Goose Neck. Complete removal was observed in 18/19 bailout procedures and in 16/17 procedures using wrap-around. A capturable free end was present in 15/19 procedures: two were completed by direct Goose Neck capture alone, four proceeded to wrap-around because direct capture failed, and nine proceeded because capture did not provide stable inferior control. Four procedures had no capturable free end and therefore began the femoral sequence with RF Marinr wrap-around. All final bailout retrievals were femoral. Median target-lead dwell time in the 17 wrap-around procedures was 8.0 years (interquartile range, 5.0–16.0 years; range, 2–27 years). The sole incomplete-removal case retained a 20-mm distal coronary-sinus lead tip that remained fixed without migration, embolization, or reintervention through day 30.

Supplementary Table S1A. Case-level comparison of all transvenous lead extraction bailout procedures (n = 19).

Case	Device; indication; target lead(s); dwell	Superior tool; femoral-escalation trigger code(s)	Case-verified femoral sequence	Dissection sheath(s); outcome / selected complexity
B01	DDD pacemaker; lead dysfunction; atrial; 7 y	Evolution RL; 2,4	Direct Goose Neck capture; control inadequate; RF Marinr wrap; Marinr-tip capture; closed loop; lead-segment recapture; femoral retrieval	9 Fr; complete
B02	DDD ICD; pocket infection; RV ICD; 2 y	Bulldog Lead Extender; 1,3	Direct Goose Neck capture; control inadequate; RF Marinr wrap; Marinr-tip capture; closed loop; lead-segment recapture; femoral retrieval	11 Fr; complete; prior failed extraction; abandoned leads
B03	CRT-D; lead dysfunction; CS/LV + RV ICD; 6 y	Manual; 3	Direct Goose Neck capture; control inadequate; RF Marinr wrap; Marinr-tip capture; closed loop; lead-segment recapture; femoral retrieval	9 + 11 Fr; complete
B04	CRT-D; pocket infection; CS/LV + RV ICD; 3 y	Evolution RL; 4	Direct Goose Neck capture failed; RF Marinr wrap; Marinr-tip capture; closed loop; lead-segment recapture; femoral retrieval	9 + 11 Fr; complete
B05	DDD pacemaker; pocket infection; RV pacing; 24 y	LLD; 4	Direct Goose Neck capture; femoral retrieval without wrap-around	11 Fr; complete
B06	CRT-D; lead dysfunction; RV ICD; 6 y	LLD; 4	No free end; RF Marinr wrap; stable inferior control; lead drawn into the femoral introducer without snare	11 Fr; complete
B07	DDD pacemaker; pocket infection; atrial + RV pacing; 8 y	Manual; 3,4	Direct Goose Neck capture; control inadequate; RF Marinr wrap; Marinr-tip capture; closed loop; lead-segment recapture; femoral retrieval	9 + 11 Fr; complete
B08	DDD ICD; pocket infection; atrial + RV ICD; 16 y	Evolution RL; 2,3	No free end; RF Marinr wrap; Marinr-tip capture; closed loop; lead-segment recapture; femoral retrieval	9 + 11 Fr; complete; abandoned leads

Case	Device; indication; target lead(s); dwell	Superior tool; femoral-escalation trigger code(s)	Case-verified femoral sequence	Dissection sheath(s); outcome / selected complexity
B09	DDD pacemaker; pocket infection; RV pacing; 23 y	Evolution RL; 1,2,3	Direct Goose Neck capture; control inadequate; RF Marinr wrap; Marinr-tip capture; closed loop; lead-segment recapture; femoral retrieval	11 Fr; complete; abandoned leads
B10	DDD pacemaker; CRT-D upgrade; atrial + RV pacing; 17 y	LLD; 3,5	Direct Goose Neck capture; control inadequate; RF Marinr wrap; Marinr-tip capture; closed loop; lead-segment recapture; femoral retrieval	9 + 11 Fr; complete
B11	VVI ICD; superior vena cava syndrome; RV ICD; 5 y	Evolution RL; 1,2,3	Direct Goose Neck capture failed; RF Marinr wrap; Marinr-tip capture; closed loop; lead-segment recapture; femoral retrieval	11 Fr; complete; venous occlusion
B12	DDD ICD; lead dysfunction; RV ICD; 9 y	Evolution RL; 4	Direct Goose Neck capture failed; RF Marinr wrap; Marinr-tip capture; closed loop; lead-segment recapture; femoral retrieval	11 Fr; complete
B13	DDD pacemaker; lead dysfunction; RV pacing; 24 y	Manual; 4	Direct Goose Neck capture; control inadequate; RF Marinr wrap; Marinr-tip capture; closed loop; lead-segment recapture; femoral retrieval	11 Fr; complete
B14	DDD pacemaker; lead dysfunction; atrial + RV pacing; 27 y	Manual; 3,4	Direct Goose Neck capture failed; RF Marinr wrap; Marinr-tip capture; closed loop; lead-segment recapture; femoral retrieval	9 + 11 Fr; complete
B15	DDD ICD; lead dysfunction + CRT upgrade; RV ICD; 4 y	Manual; 4	Direct Goose Neck capture; control inadequate; RF Marinr wrap; Marinr-tip capture; closed loop; lead-segment recapture; femoral retrieval	11 Fr; complete
B16	DDD ICD; lead dysfunction; RV ICD; 9 y	Evolution RL; 2,4	Direct Goose Neck capture; control inadequate; RF Marinr wrap; Marinr-tip capture; closed loop; lead-segment recapture; femoral retrieval	11 Fr; complete
B17	CRT-D; lead dysfunction; RV ICD; 10 y	Manual; 4	No free end; RF Marinr wrap; Marinr-tip capture; closed loop; lead-segment recapture; femoral retrieval	11 Fr; complete
B18	DDD ICD; lead dysfunction; RV ICD; 10 y	Manual; 4	Direct Goose Neck capture; femoral retrieval without wrap-around	11 Fr; complete
B19	DDD pacemaker; lead dysfunction; CS/LV; 2 y	Manual; 4	No free end; RF Marinr wrap; Marinr-tip capture; closed loop; lead-segment recapture; femoral retrieval	9 Fr; clinical success; stable 20-mm distal CS tip

Note: Case labels are arbitrary and are not hospital identifiers. Trigger codes: 1, sheath non-progression; 2, loss of a stable coaxial rail; 3, deformation/elongation or impending fracture; 4, fracture with loss of superior distal-segment control; 5, further superior traction judged unsafe. Multiple codes could coexist. All procedures used the right femoral vein and a 10-Fr venous introducer; all final bailout retrievals were femoral without concurrent superior-sheath advancement. In all 17 wrap-around procedures, the RF Marinr loop was formed in the free right atrium above the tricuspid valve. Each lead's dwell was calculated from its own implantation year; simultaneously implanted target leads had identical dwell. Pocket-infection cases were not classified as endocarditis: cultures were obtained when systemic involvement was suspected, and no vegetation was documented. No patient had prior sternotomy. Calcification was not systematically graded. LLD, Lead Locking Device; CS/LV, coronary-sinus (left-ventricular); RV, right ventricular.

No consensus-defined major or minor complication was documented through day 30. Four minimal subcutaneous pocket/incisional hematomas required observation only and are reported separately in Table S4. The single retained fragment was a 20-mm distal coronary-sinus (left-ventricular) lead tip fixed in a distal coronary-sinus branch; it remained unchanged on procedural fluoroscopy and on day-30 chest radiography and fluoroscopy, without migration, embolization, or reintervention. Abbreviations: CS, coronary sinus; LV, left ventricular; RV, right ventricular.

This table is descriptive. Small counts, overlapping target-lead categories, and operator-selected escalation preclude comparative statistical inference or attribution of outcome to an individual bailout component.

Supplementary Table S1B. Case-verified stepwise procedural decision framework.

Stage	Decision point	Case-verified action	Mechanical objective
Superior preparation	Initial pathway	Release the fixation sleeve; retract an active helix when feasible; introduce a locking device; reinforce the lead; apply controlled traction	Establish axial control while preserving lead integrity
Mechanical dissection	Traction insufficient	Use a target-adapted sheath: 9 Fr for atrial or CS/LV leads; 11 Fr for RV pacing or shock-coil ICD leads	Dissect adhesions while maintaining a coaxial rail
Superior-pathway check	Stable coaxial rail and controlled progress	Continue superior dissection	Complete extraction without femoral escalation
Femoral escalation	Any trigger 1–5	Obtain right femoral venous access through a 10-Fr introducer and assess for a capturable free lead end	Restore controlled inferior access
Direct Goose Neck	Free end present (15/19)	Attempt direct capture with the 15-mm Amplatz Goose Neck snare; adequate control permitted direct femoral retrieval in 2/15	Secure the target for inferior retrieval
RF Marinr wrap-around	No free end (4/19), failed capture (4/15), or inadequate direct control (9/15)	Form a loop in the free right atrium above the tricuspid valve	Encircle the lead and establish controlled inferior purchase
Combined sequence	Goose Neck used after wrap-around (16/17)	Capture the Marinr distal tip; close the loop; mobilize the lead; recapture the freed lead segment	Convert loop control into definitive femoral retrieval
Wrap-only sequence	No Goose Neck after wrap-around (1/17)	Use stable Marinr control to draw the lead into the 10-Fr femoral introducer	Complete femoral retrieval without snare
Final extraction	Inferior control established	Retrieve femorally with inferior traction; do not advance the superior sheath concurrently	Complete the bailout pathway

Note: The framework summarizes the actual sequence confirmed by review of procedural reports and fluoroscopic images. It was applied clinically without a fixed time, force, or attempt threshold and has not been prospectively validated.

This table summarizes the case-verified procedural sequence: superior preparation and target-adapted dissection-sheath use; assessment of rail stability and controlled progress; right-femoral escalation through a 10-Fr introducer when any qualitative trigger was present; direct 15-mm Amplatz Goose Neck capture when a free end was available; RF Marinr right-atrial wrap-around when no free end was available or direct capture failed or remained mechanically inadequate; and final femoral retrieval. In the 16 combined procedures, the Goose Neck captured the Marinr distal tip

to close the loop and subsequently recaptured the freed lead segment. One wrap-around-only procedure was completed without a snare. A Needle's Eye Snare was not used.

3. Temporal and Safety Descriptions

Supplementary Table S2. Annual distribution of the included analytic cohort and observed outcomes, 2014–2025.

Year	Procedures, n	Bailout, n	Wrap-around, n	Complete procedural success, n
2014	2	1	0	2
2015	8	1	1	8
2016	5	0	0	5
2017	5	0	0	5
2018	17	4	4	17
2019	8	5	5	8
2020	7	4	4	7
2021	8	0	0	8
2022	4	1	0	4
2023	3	0	0	3
2024	1	1	1	1
2025	10	2	2	9

Bailout was defined as femoral snaring and/or wrap-around. The annual counts sum to the 78-patient analytic cohort and are not total center-wide TLE volume. Included-cohort counts ranged from 1 to 17 per year and varied without a consistent monotonic pattern; the early and contemporary eras contained 45 (7.5/year) and 33 (5.5/year) included procedures, respectively. These counts do not establish a center-volume or learning trend. The center is a small, single-operator program performing approximately 10 TLE procedures per year. Following an archival-system change, complete center-wide year-by-year counts for 2014–2025 were unavailable. The first recorded wrap-around procedure occurred in 2015. The Evolution Mechanical Dilator Sheath Set was used in 2014 and the Evolution RL Controlled-Rotation Dilator Sheath Set from 2015 onward.

Supplementary Table S3. Descriptive early-versus-contemporary era summary.

Era	Procedures, n	Target-lead dwell time, years	Bailout, n/N (%)	Wrap-around, n/N (%)	Complete success, n/N (%)	Bailout success, n/N (%)
2014–2019	45	Median: 4; IQR: 2–8	11/45 (24.4)	10/45 (22.2)	45/45 (100.0)	11/11 (100.0)
2020–2025	33	Median: 6; IQR: 3–8	8/33 (24.2)	7/33 (21.2)	32/33 (97.0)	7/8 (87.5)

The equal six-year split was prespecified for description and included in the exploratory Firth model. Similar crude bailout proportions across eras and an imprecise adjusted era estimate cannot identify learning, tool-availability, referral, or escalation-threshold effects.

Supplementary Table S4. Major and minor safety outcomes by prespecified follow-up window.

Outcome	During procedure	Postprocedural / in-hospital	Post-discharge through day 30	Cumulative through day 30
**Patients assessed**	78/78	78/78	74/74 eligible	78/78
**All-cause mortality**	0/78	0/78	0/74	0/78
**Procedure-related mortality**	0/78	0/78	0/74	0/78
**Cardiovascular mortality**	0/78	0/78	0/74	0/78
**Noncardiovascular/infection-related mortality**	0/78	0/78	0/74	0/78
**Major complications**	0/78	0/78	0/74	0/78
**Minor complications**	0/78	0/78	0/74	0/78
**Emergency surgical/endovascular intervention**	0/78	0/78	0/74	0/78
Retained-fragment migration or embolization	0/1	0/1	0/1	0/1
Rehospitalization	—	—	0/74	0/78
Repeat intervention or surgery	—	—	0/74	0/78
Temporary pacing support	—	4/8 pacemaker pocket-infection cases	—	4/8 pacemaker pocket-infection cases
Permanent reimplantation pathway	47/78 same-session permanent reimplantation	30 infection cases followed a delayed strategy; timing by window was not recorded	1 SVCS case underwent subsequent-session implantation; timing by window was not recorded	47 same-session; 31 delayed/subsequent-session pathways; see Table S5B
Length of stay	—	Median: 1 day; IQR: 1–3; range: 1–45 (n = 78)	—	—
Persistent/recurrent bacteremia	—	0/30 infection	0/26 infection	0/30 infection
Recurrent pocket/systemic CIED infection	—	0/30 infection	0/26 infection	0/30 infection
Observation-only minimal subcutaneous pocket/incisional hematoma	Exact first-detection window not separately recorded	Exact first-detection window not separately recorded	Detected during wound surveillance through day 30	4/78; all primary-only (1 same-session reimplantation, 3 extraction without same-session reimplantation)

Outcome	During procedure	Postprocedural / in-hospital	Post-discharge through day 30	Cumulative through day 30
Hematoma requiring medical or procedural treatment	0/4	0/4	0/4	0/4
Retained-fragment reintervention	0/1	0/1	0/1	0/1

Events were counted in the first window in which they became evident and in the cumulative 30-day assessment. Length of stay is reported in the postprocedural/in-hospital column because it is not a cumulative event. Major complications were life-threatening or fatal, caused persistent/significant disability, prolonged hospitalization, or required major surgical/endovascular intervention. Minor complications required medical treatment or a minor procedure. The four minimal hematomas required observation only and therefore appear as wound findings rather than formal minor complications. Four endocarditis patients remained hospitalized through day 30, leaving 74 patients with a post-discharge interval. Infection recurrence is reported from extraction through day 30, not as a post-reimplantation rate.

4. Data Availability for Procedural and Complexity Variables

Supplementary Table S5. Availability and analytical handling of procedural and complexity variables.

Variable	Data availability and analytical handling
Target-lead implantation year and dwell	Available for 78/78 and calculated as extraction year minus implantation year of the procedural target lead. In bailout procedures, the value belongs to the lead prompting femoral escalation. When two target leads were present, both had the same implantation year and identical dwell time.
Case-level device order	Available for all 19 bailout procedures after review of procedural reports and fluoroscopic images; reported in Table S1A.
Escalation trigger and wrap-around mechanics	Available for all 19 bailout procedures. Trigger codes, capturable-free-end status, direct-capture result, free-right-atrial loop location, combined closed-loop sequence, and final femoral retrieval are reported in Tables S1A–S1B and Figure 2.
Procedure duration, fluoroscopy, and air kerma	Extraction-phase duration, total skin-to-skin duration, post-extraction interval, extraction fluoroscopy, and fluoroscopy-system-reported extraction air kerma were available for 78/78. Results are reported in Table S5A and Supplementary Figure S1. Total-case fluoroscopy could not be reconciled reliably, and directly measured total-case air kerma was unavailable.

Variable	Data availability and analytical handling
Anesthesia conversion, blood loss/transfusion, length of stay, and discharge timing	Initial local anesthesia with procedural sedation was recorded in 78/78 procedures; conversion to general anesthesia and transfusion were each 0/78. Median length of stay was 1 day (IQR, 1–3; range, 1–45; n = 78). By indication, the median was 1 day for noninfection (IQR, 1–1; range, 1–1), 3 days for pocket infection (IQR, 3–3.75; range, 3–14), and 42 days for endocarditis (IQR, 37.5–44.25; range, 30–45). Estimated blood loss and exact discharge dates were not recorded.
Unplanned surgical or percutaneous support	Unplanned surgical support 0/78, unplanned percutaneous support 0/78, and vascular complications 0/78.
Sheath strategy	All bailout procedures used a right-femoral 10-Fr introducer. Mechanical dissection sheaths were 9 Fr for atrial and coronary-sinus (left-ventricular) leads and 11 Fr for right-ventricular pacing or shock-coil ICD leads; patient-level selections are reported in Table S1A.
Lead model, fixation mechanism, and ICD coil configuration	Not recoverable because all index implants were performed at outside institutions; these characteristics were not inferred.
Selected bailout complexity characteristics	Among 19 bailout procedures, 6 involved pocket infection, 13 were noninfectious, and none involved an endocarditis indication. Venous occlusion was documented in 1/19, abandoned leads in 3/19, a previous unsuccessful extraction in 1/19, and prior sternotomy in 0/19. No vegetation was documented in the pocket-infection bailout cases. Calcification was not systematically graded.
Validated extraction-complexity score	Not calculated because several required components were unavailable.
Evolution RL introduction	The Evolution Mechanical Dilator Sheath Set was used in 2014 and the Evolution RL Controlled-Rotation Dilator Sheath Set from 2015 onward.
Retained coronary-sinus fragment	The retained material was a 20-mm distal coronary-sinus (left-ventricular) lead tip fixed in a distal coronary-sinus branch. It remained stable on procedural and day-30 imaging without migration, embolization, or reintervention.
Analytic-cohort flow	Records were retrievable for 82 procedures; four image-only records lacked clinical history, detailed procedural/material information, and 30-day follow-up, leaving 78 patients for analysis.
Center TLE volume	The center performs approximately 10 TLE procedures per year. Complete center-wide annual counts for 2014–2025 were unavailable after archival-system changes.

Variable	Data availability and analytical handling
Thirty-day clinical follow-up	Study-center records were available for 78/78. The protocol comprised predischarge assessment, wound/pocket review at 48–72 hours and 7–10 days, and device interrogation at approximately 4 weeks. Formal major/minor complications were 0/78; four minimal observation-only hematomas are reported separately. Four endocarditis patients remained hospitalized through day 30.
Previous extraction attempt	One prior unsuccessful extraction was documented: the generator had been removed and intact disconnected leads were left in the pocket. No other prior attempt was documented in the available records.

Unavailable case-level clinical or lead-design characteristics were not reconstructed from indirect indicators. Records were retrievable for 82 procedures, and four image-only records were excluded because the clinical history, detailed procedural/material documentation, and follow-up required for analysis were unavailable. The 78-patient cohort had complete duration endpoints and extraction-phase fluoroscopy/air-kerma data. Supplementary Table S5A and Supplementary Figure S1 report these resource outcomes; Supplementary Table S5B reports reimplantation and length of stay.

Supplementary Table S5A. Procedural duration, extraction fluoroscopy, and fluoroscopy-system-reported air kerma by procedural pathway.

Metric	Overall (n = 78)	Primary only (n = 59)	Bailout (n = 19)	P value
Extraction-phase duration, min	Median: 79.7; IQR: 58.3–111.4; range: 24–216	Median: 71.0; IQR: 58.0–106.5; range: 24–179	Median: 93.8; IQR: 77.5–119.8; range: 33–216	0.249
Total skin-to-skin duration, min	Median: 110.1; IQR: 82.6–148.7; range: 39–271	Median: 108.4; IQR: 80.0–134.7; range: 39–214	Median: 126.0; IQR: 100.4–155.1; range: 47–271	0.222
Post-extraction to final closure, min	Median: 32.5; IQR: 17.1–39.7; range: 10–64.9	Median: 32.5; IQR: 17.3–38.2; range: 13–55.7	Median: 32.0; IQR: 16.9–44.3; range: 10–64.9	0.422
Extraction fluoroscopy, min	Median: 21.8; IQR: 6.9–35.5; range: 2.0–76.0	Median: 16.6; IQR: 5.8–29.1; range: 2.0–72.4	Median: 31.4; IQR: 21.4–43.3; range: 4.0–76.0	0.011
Extraction air kerma, mGy	Median: 324.5; IQR: 125.6–1036.8; range: 55.0–3624.0	Median: 346.4; IQR: 187.5–721.1; range: 55.0–3624.0	Median: 282.2; IQR: 108.0–1205.9; range: 66.8–2700.0	0.736

Values are reported as the median, interquartile range (IQR), and observed range. Extraction-only metrics exclude same-session implantation. P values compare primary-only versus bailout procedures using two-sided exploratory Mann–Whitney U tests. Wrap-around occurred in 17/19 bailout procedures and was not analyzed as an independent comparison group.

Supplementary Table S5B. Reimplantation pathway and length of stay.

Item	Numerator/denominator	Summary	Data status
Same-session permanent reimplantation	47/78	All noninfection	Patient-level
Lead dysfunction without CRT-upgrade code	36/47	Same-session indication	Patient-level indication code
Lead dysfunction + CRT upgrade	2/47	Same-session indication	Patient-level indication code
CRT-D upgrade without lead-dysfunction code	4/47	Same-session indication	Patient-level indication code
Other same-session indications	5/47	3 lead-related tricuspid valve dysfunction; 1 repositioning; 1 pericardial penetration	Patient-level indication code

Item	Numerator/denominator	Summary	Data status
Infection cases with same-session permanent reimplantation	0/30	All 30 subsequently followed delayed permanent reimplantation after culture review and completion of prescribed antimicrobial treatment; exact dates were not recorded	Patient-level same-session status + verified delayed pathway
Temporary pacing support	4/8	Four pacemaker-dependent pocket-infection patients	Verified cohort count
Same-session total skin-to-skin duration	47/47	Median: 126.0 min; IQR: 100.9–156.0; range: 59.6–271.0	Patient-level
Post-extraction to final closure with same-session reimplantation	47/47	Median: 38.4 min; IQR: 33.5–43.6; range: 24.2–64.9	Arithmetic derivation
Length of stay, overall	78/78	Median: 1 day; IQR: 1–3; range: 1–45	Hospital records
Length of stay, noninfection	48/48	Median: 1 day; IQR: 1–1; range: 1–1	Hospital records
Length of stay, CIED pocket infection	26/26	Median: 3 days; IQR: 3–3.75; range: 3–14	Hospital records
Length of stay, endocarditis	4/4	Median: 42 days; IQR: 37.5–44.25; range: 30–45	Hospital records
Delayed infection reimplantation detail	30/30 pathway; 4/30 side confirmed	After negative cultures, the four pacemaker-dependent patients underwent permanent-device reimplantation in the right pectoral region, contralateral to the infected pocket; exact dates and remaining side/device details were not recorded	Verified aggregate; exact dates were not recorded
Noninfection superior vena cava syndrome case without same-session reimplantation	1/1	Computed tomography venography was performed; pocket infection, bacteremia, and endocarditis were excluded; a permanent device was implanted in a subsequent session	Verified pathway; exact date, side, and device type were not recorded
Post-extraction to final closure without same-session reimplantation	31/31	Median: 16.0 min; IQR: 14.5–17.8; range: 10–23.4	Patient-level

The post-extraction interval equals total skin-to-skin duration minus extraction-phase duration. Temporary pacing is not classified as same-session permanent reimplantation. Exact dates or device details unavailable at case level are stated explicitly.

5. Exploratory Penalized Regression and Sensitivity Analyses

With 19 bailout events, the primary exploratory Firth logistic model included three clinically selected predictors specified before model fitting: patient-level target-lead dwell time, calendar era, and number of targeted leads. A sensitivity model replaced targeted-lead count with any targeted ICD or coronary-sinus lead. A custom Python implementation maximized the Jeffreys-prior penalized likelihood; profile penalized-likelihood confidence intervals and penalized likelihood-ratio P values are reported. Neither model is causal.

Supplementary Table S6. Exploratory Firth logistic models for any bailout use.

Model	Covariate	Adjusted OR (95% CI)	P
Primary	Target-lead dwell time, per year	1.14 (1.05–1.25)	<0.001
Primary	Contemporary era (2020–2025 vs 2014–2019)	1.12 (0.36–3.48)	0.848
Primary	Number of targeted leads, per additional lead	1.48 (0.72–3.14)	0.283
Sensitivity	Target-lead dwell time, per year	1.16 (1.07–1.29)	<0.001
Sensitivity	Contemporary era (2020–2025 vs 2014–2019)	1.09 (0.35–3.43)	0.875
Sensitivity	Any targeted ICD or coronary-sinus lead	1.96 (0.53–9.69)	0.331

ORs are adjusted within each listed model. The sensitivity complexity marker replaced targeted-lead count; it was not added as a fourth predictor. CI, profile penalized-likelihood confidence interval; ICD, implantable cardioverter-defibrillator; OR, odds ratio.

Supplementary Table S7. Primary-model diagnostics and robustness checks.

Check	Result
Model convergence	Converged in 8 iterations
Maximum leverage	0.184
Adjusted quadratic dwell term	Penalized likelihood-ratio P = 0.499
Leave-one-out dwell-time OR range	1.13–1.17 across 78 refits
Events and clinically selected model predictors	19 events; 3 predictors (6.3 events per predictor)

The Firth modified score was numerically checked against a finite-difference gradient. These checks address small-sample estimation and single-observation sensitivity but do not compensate for integer calendar-year resolution or other unrecorded complexity factors.

6. Procedural Resource Visualization

Supplementary Figure S1. Extraction resource use by procedural pathway

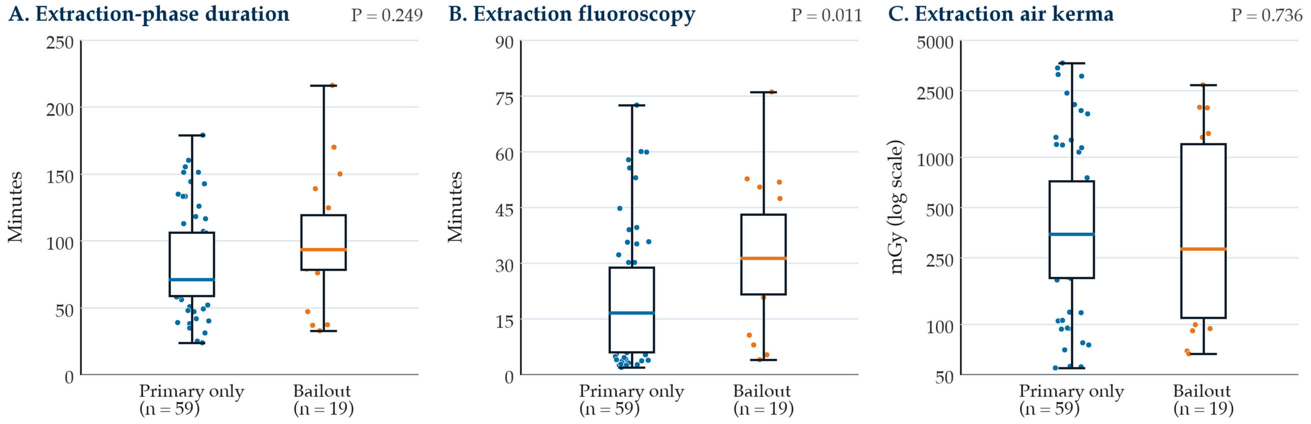

Supplementary Figure S1. Extraction resource use by procedural pathway. Individual patient observations and boxplots are shown for primary-only (n = 59) and bailout (n = 19) procedures; boxes indicate the interquartile range, center lines the median, and whiskers the observed range. Air kerma is displayed on a logarithmic scale. P values are from exploratory two-sided Mann–Whitney U tests. Extraction-phase duration ends with final target-lead removal, and fluoroscopy/air kerma exclude same-session implantation.
